# Profiling the gut microbiota to assess infection risk in *Klebsiella pneumoniae*-colonized patients

**DOI:** 10.1080/19490976.2025.2468358

**Published:** 2025-02-18

**Authors:** Flavio De Maio, Delia Mercedes Bianco, Giulia Santarelli, Roberto Rosato, Francesca Romana Monzo, Barbara Fiori, Maurizio Sanguinetti, Brunella Posteraro

**Affiliations:** a Department of Laboratory and Hematological Sciences, Fondazione Policlinico Universitario A. Gemelli IRCCS, Rome, Italy; b Microbiota Analysis & Microbial WGS Research Core Facility, GSTeP, Fondazione Policlinico Universitario A. Gemelli IRCCS, Rome, Italy; c Department of Infectious Diseases, Castle Hill Hospital, East Riding of Yorkshire, Cottingham, UK; d Precision Medicine in Clinical Microbiology Unit, Direzione Scientifica, Fondazione Policlinico Universitario A. Gemelli IRCCS, Rome, Italy

**Keywords:** Gut microbiota, genotype, colonization

## Abstract

Vornhagen et al. introduced a model combining gut microbiota structure and *Klebsiella pneumoniae* genotype to assess infection risk in *K. pneumoniae*-colonized patients. Building on their findings, we investigated the gut microbiota composition and *K. pneumoniae* genotype in 16 colonized patients, five of whom had bloodstream infections at the time of fecal sampling. Importantly, we did not apply the original machine learning model due to the small sample size of our cohort. Instead, we explored the distribution of key antimicrobial resistance and stress resistance genes and analyzed gut community structure based on amplicon sequence variants (ASVs) of the V3–V4 16S rRNA region. Notably, distinct gene profiles were observed in both infected and non-infected patients, and three patients without bloodstream infections showed no detectable *Klebsiella* ASVs despite microbiological confirmation of colonization. These findings highlight the need to integrate gut microbiota composition data into infection risk assessment and address limitations in taxonomic resolution and sample size. Future studies should aim to develop streamlined tools for clinical application in *K. pneumoniae*-colonized patients.

Dear Editor,

We recently analyzed the study by Vornhagen et al. (2024) published in *mSystems*,^1^ which provides valuable insights into the role of gut microbiota in modulating infection risk in patients colonized with *Klebsiella pneumoniae*. The study demonstrated that integrating gut microbiota data with *K. pneumoniae* genotype improves the prediction of infection risk, presenting promising avenues for infection control and prevention strategies.

Building on the findings of Vornhagen et al.,^1^ we sought to explore variables potentially contributing to infection risk in *K. pneumoniae*-colonized patients by analyzing the gut microbiota composition and the *K. pneumoniae* genotype in a small cohort. Our study included 16 patients identified through active surveillance for carbapenem-resistant *K. pneumoniae* at a single large teaching hospital in Rome, Italy. Rectal swabs were collected to confirm *K. pneumoniae* colonization using selective culture methods, and fecal samples were obtained within 18–24 hours of the positive rectal screening for gut microbiota analysis. Of the 16 patients, five (31.2%) had a bloodstream infection at the time of fecal sampling. This cohort allowed us to investigate the interplay between gut microbiota structure and the genetic profiles of *K. pneumoniae* isolates. However, given the small sample size, we focused on describing these variables and their potential implications for infection risk rather than testing predictive models, consistent with the exploratory nature of this study.

Our study focused on exploring the set of 27 *Klebsiella* genes, including antimicrobial resistance (AMR) and non-AMR (e.g., stress resistance) genes, previously identified by Vornhagen et al.^2^ as being associated with infection in *K. pneumoniae*-colonized patients. Among these, two of the five genes validated in a geographically independent cohort (*aac(6’)-Ib-cr5* and *bla*_CTX-M-15_) were detected in our isolates (Figure 1). The *aac(6’)-Ib-cr5* gene, encoding an aminoglycoside acetyltransferase active against fluoroquinolones, was present in 12.5% (2/16) of the isolates, one of which was from a patient with bloodstream infection. The *bla*_CTX-M-15_ gene, encoding a broad-spectrum β-lactamase, was detected in 75% (12/16) of the isolates, including four from patients with bloodstream infections. While the presence of these genes highlights their potential importance in understanding infection risk, their distribution among both infected and non-infected patients underscores the limitations of genotype alone in predicting infection outcomes.

**Figure 1.**
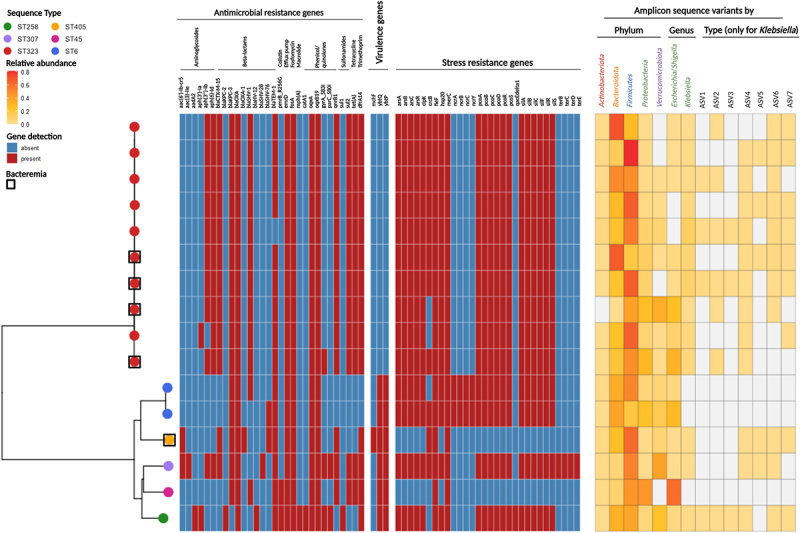
Gene content profiles of 16 gut-colonizing *Klebsiella pneumoniae* isolates, categorized by sequence types (STs), alongside the gut microbiota composition features of patients sampled within 18–24 hours after positive rectal swabs. The phylogenetic relationships among isolates are depicted as a neighbor-joining tree, generated based on whole genome multilocus sequence typing (Ridom SeqSphere+; https://www.Ridom.de/seqsphere). Each cluster includes isolates with specific antimicrobial resistance, virulence, and stress resistance genes, as annotated using AMRFinderPlus (https://github.com/ncbi/amr) and ABRicate (https://github.com/tseemann/abricate) tools. The whole genome sequencing confirmed the species identification and revealed six distinct STs among the isolates: ST323 (10 isolates), ST6 (2 isolates), ST45 (1 isolate), ST258 (1 isolate), ST307 (1 isolate), and ST405 (1 isolate). Chromosomal phylogeny showed that ST323 and ST6 formed distinct clusters, designated as Cluster I and Cluster II, respectively, while ST45, ST258, and ST307 grouped together. ST405 was closer to Cluster II than Cluster I and was isolated from a patient with a bloodstream infection. For gut microbiota analysis, amplicon sequence variants (ASVs) of the V3–V4 16S rRNA gene region were stratified, and *Klebsiella*-specific ASVs were analyzed. Patients with bloodstream infections had between two and six *Klebsiella* ASVs, while three patients without bloodstream infections had no detectable *Klebsiella* ASVs. Further methodological details are provided in the supplementary file accompanying this article.

The gut community structure – particularly in terms of *K. pneumoniae* dominance and the interactions with other bacterial species – plays a crucial role in the colonization process. Building on the findings of Vornhagen et al.,^1^ we analyzed the gut microbiota composition in 16 *K. pneumoniae*-colonized patients to explore variables potentially relevant for infection risk assessment. Our approach focused on the identification of amplicon sequence variants (ASVs) targeting the V3–V4 16S rRNA gene regions. However, we acknowledge that the use of 16S rRNA gene sequencing, particularly at the V3–V4 region, has intrinsic limitations in taxonomic resolution and cannot definitively distinguish *K. pneumoniae* from other members of the *Klebsiella* genus. Consistent with these limitations, the *Klebsiella* ASVs detected in our study may represent different *Klebsiella* species, as also observed in the study by Vornhagen et al.^1^

In our cohort (Figure 1), we detected seven *Klebsiella* ASVs (designated ASV1 to ASV7), with 81.2% (13/16) of patients harboring between two and six *Klebsiella* ASVs. Interestingly, 18.8% (3/16) of the patients had no detectable *Klebsiella* ASVs, despite microbiological confirmation of colonization. None of these three patients developed bloodstream infections at the time of sampling. This highlights that, although 16S rRNA gene sequencing provides an overview of gut community structure, it may not be sensitive enough to capture low-abundance *Klebsiella* species in fecal samples. Our findings emphasize the importance of integrating genomic and microbiota data to better characterize colonization dynamics and their association with infection risk. Additionally, quantitative PCR (qPCR) could offer higher sensitivity for detecting *K. pneumoniae* and may help overcome the limitations associated with 16S rRNA gene sequencing in future studies.

The resident gut microbiota can limit the colonization of *K. pneumoniae* and other opportunistic pathogens by modulating the host immune response through a consortium of *Bacteroidota*.^3^ However, in the gut environment, *K. pneumoniae* can bypass microbiota-mediated colonization resistance by metabolizing alternative nutrient sources, such as fucose, which is released from mucins by enzymes produced by *Bacteroides*.^4^ In our study, *Bacteroidota* and *Firmicutes* were the most abundant gut microbiota phyla, consistent with their role as core members of the gut microbiome. While this finding does not imply an exclusive interaction between these phyla and *K. pneumoniae*, it suggests that *K. pneumoniae* colonization and persistence may rely on a broader interplay involving key microbial species and the host environment. The balance between *Bacteroidota* and *Firmicutes*—as reflected by the *Firmicutes*-to-*Bacteroidota* ratio – has been recognized as a significant parameter influencing the colonization of opportunistic pathogens.

The protective role of specific gut microbiota members against *K. pneumoniae* can be offset by bacterial factors that enhance gut fitness, such as the factors encoded by AMR and non-AMR genes. One example is the *terC* gene, which was detected in the ST307 isolate in our cohort (Figure 1). This gene has been associated with increased gut fitness in the presence of microbiota species that produce short-chain fatty acids (SCFAs), which are important gut metabolites.^5^ These findings underscore the complexity of interactions between the gut microbiota and *K. pneumoniae*, highlighting the need for future studies to further elucidate the role of microbiota-derived metabolites and bacterial genetic determinants in colonization and infection risk.

The study by Vornhagen et al.,^1^ situated within the broader research conducted by the same authors on patient factors, gut dominance, and *K. pneumoniae* genotype,^2,6,7^ marks a significant advance in understanding infection risk in *K. pneumoniae*-colonized patients. By integrating multiple datasets, this study offers a comprehensive framework for developing predictive models. However, while it establishes a solid foundation, there remains room for improvement in translating these models into actionable clinical tools. Our findings, in line with this effort, underscore the importance of evaluating microbiota structure and its interactions with *K. pneumoniae* genotype. Nevertheless, the small cohort size in our study limits the extent to which our conclusions can be generalized. Future research should aim to refine these models by incorporating microbiota insights and reducing the number of predictive variables to develop streamlined, clinically manageable tools that can be readily applied in patient care.

## Supplementary Material

Supplemental Material

## References

[1] Vornhagen J, Rao K, Bachman MA, Chia N. Gut community structure as a risk factor for infection in Klebsiella pneumoniae-colonized patients. mSystems. 2024;9(8):e0078624. doi:10.1128/msystems.00786-24. 38975759 PMC11334466

[2] Vornhagen J, Roberts EK, Unverdorben L, Mason S, Patel A, Crawford R, Holmes CL, Sun Y, Teodorescu A, Snitkin ES, et al. Combined comparative genomics and clinical modeling reveals plasmid-encoded genes are independently associated with Klebsiella infection. Nat Commun. 2022;13(1):4459. doi:10.1038/s41467-022-31990-1. 35915063 PMC9343666

[3] Bray AS, Zafar MA, Ottemann KM. Deciphering the gastrointestinal carriage of Klebsiella pneumoniae. Infect Immun. 2024;92(9):e0048223. doi:10.1128/iai.00482-23. 38597634 PMC11384780

[4] Hudson AW, Barnes AJ, Bray AS, Ornelles DA, Zafar MA, Raffatellu M. Klebsiella pneumoniae _L_-fucose metabolism promotes gastrointestinal colonization and modulates its virulence determinants. Infect Immun. 2022;90(10):e0020622. doi:10.1128/iai.00206-22. 36129299 PMC9584338

[5] Vornhagen J, Bassis CM, Ramakrishnan S, Hein R, Mason S, Bergman Y, Sunshine N, Fan Y, Holmes CL, Timp W, et al. A plasmid locus associated with Klebsiella clinical infections encodes a microbiome-dependent gut fitness factor. PloS Pathog. 2021;17(4):e1009537. doi:10.1371/journal.ppat.1009537. 33930099 PMC8115787

[6] Rao K, Patel A, Sun Y, Vornhagen J, Motyka J, Collingwood A, Teodorescu A, Baang JH, Zhao L, Kaye KS, et al. Risk factors for Klebsiella infections among hospitalized patients with preexisting colonization. mSphere. 2021;6(3):e0013221. doi:10.1128/mSphere.00132-21. 34160237 PMC8265626

[7] Sun Y, Patel A, SantaLucia J, Roberts E, Zhao L, Kaye K, Rao K, Bachman MA, D’Orazio SEF. Measurement of Klebsiella intestinal colonization density to assess infection risk. mSphere. 2021;6(3):e0050021. doi:10.1128/mSphere.00500-21. 34160234 PMC8265666

