# Supplementary material for: Profiling the gut microbiota to assess infection risk in Klebsiella pneumoniae-colonized patients
Source: Gut Microbes. 2025 Feb 18;17(1):2468358. doi: 10.1080/19490976.2025.2468358 (PMC11845061; doi:10.1080/19490976.2025.2468358)
Supplement: Supplemental Material [file KGMI_A_2468358_SM0671.docx]

**Supplementary Methods**

**Fecal Samples and Bacterial Isolates**

Rectal swab samples were collected as part of active surveillance for carbapenem-resistant *Klebsiella pneumoniae* in hospitalized patients. Upon receipt at the Clinical Microbiology Laboratory of the Fondazione Policlinico Universitario A. Gemelli IRCCS in Rome, Italy, samples were cultured on CHROMID CARBA SMART selective chromogenic medium for carbapenemase-producing organisms (bioMérieux, Mary l’Étoile, France). Positive cultures prompted the collection of fecal samples within 18–24 hours to confirm colonization. Fecal samples were divided for microbiological testing on bioMérieux MacConkey agar and gut microbiota analysis. *K. pneumoniae* isolates were identified using MALDI-TOF mass spectrometry and tested for carbapenem resistance via routine antimicrobial susceptibility testing analysis.

Ethical approval was deemed unnecessary for this study, given its purely descriptive, exploratory, and observational nature, with no implications for patient management. This determination was supported by the following considerations: (i) all samples used in the study were residual aliquots collected in completely anonymized vials or archived bacterial isolates obtained during routine clinical laboratory operations, and (ii) no access to personal patient data was permitted.

**Whole-Genome Sequencing Analysis**

DNA was extracted from rectal swab *K. pneumoniae* isolates using the DANAGENE Microbial DNA kit (Danagen-Bioted, Barcelona, Spain). DNA purity and concentration were evaluated with a NanoDrop One spectrophotometer (Thermo Fisher, Waltham, MA, USA). Libraries were prepared using the Illumina DNA Prep kit (Illumina, San Diego, CA, USA) and sequenced in a paired-end protocol (PE250) on the Illumina MiSeq DX platform, following the manufacturer’s recommendations as previously described [1]. Quality control of raw reads was performed with Fastp (https://github.com/OpenGene/fastp), and de novo assembly was carried out using Unicycler (https://github.com/rrwick/Unicycler). Species identification and contamination screening were conducted using the CLC Genomic Workbench software (https://www.qiagen.com/us/products/discovery-and-translational-research/next-generation-sequencing/informatics-and-data/analysis-and-visualization/clc-genomics-workbench). The isolates’ pangenome consisted of 13,004 total annotations, with 3385 genes belonging to the core genome (≥95% of genomes) and 9619 genes to the accessory genome (<95% of genomes). Antimicrobial resistance genes and bacterial virulence factors were annotated with AMRFinderPlus (https://github.com/ncbi/amr) and their presence was cross-verified using ABRicate (https://github.com/tseemann/abricate), which uses the bacterial virulence factor database (VFDB, http://www.mgc.ac.cn/VFs/). Sequence types were determined using multilocus sequence typing (MLST), and core-genome MLST profiles were generated with the Ridom SeqSphere+ software (https://www.ridom.de/seqsphere), based on 2358 core genes. All isolates were classified as the classical pathotype, as no genes for well-known virulence factors—such as *iroBCDN* (salmochelin), *iucA* (aerobactin), and *rmpA/rmpA2* (hypermucoid regulation)—were detected.

**Gut Microbiota Analysis**

DNA from fecal samples was extracted using the DANAGENE MICROBIOME Fecal DNA kit under sterile conditions [2]. Approximately 200 mg of sample was homogenized in cetyltrimethylammonium bromide buffer, and DNA was extracted and then checked for purity and concentration, as specified above. Libraries targeting the V3–V4 hypervariable regions of the 16S ribosomal RNA gene were prepared and sequenced on the Illumina MiSeq DX platform. DNA amplification was performed by PCR using the following primers: V3_Next_For, 5’-TCGTCGGCAGCGTCAGATGTGTATAAGAGACAGCCTACGGGNGGCWGCAG-3’ and V4_Next_Rev, 5’-TCTCGTGGGCTCGGAGATGTGTATAAGAGACAGGACTACHVGGGTATCTAATCC-3’), which included sequences specific for Nextera transposons and BV5/AV6 priming sites [3]. Amplified DNA was purified using AMPure XP beads, quantified, and indexed for sequencing. Amplicons were then pooled in equimolar concentrations and sequenced using a 2 × 300 paired-end protocol.

Raw sequences were processed with QIIME2 (v.2023.5.1). Reads were demultiplexed, adapters were trimmed with the “cutadapt trim-paired” plugin, and denoising was performed using DADA2. Taxonomic classification of ASVs was conducted using VSEARCH with the SILVA 138 database at a 99% similarity threshold. Unassigned ASVs and those with a prevalence <1% were excluded. Taxonomic relative abundances were calculated at the phylum and genus levels, and the Wilcoxon-Mann-Whitney test was used to assess statistical significance (p < 0.05). The relative abundance of *K. pneumoniae*-specific ASVs was evaluated.

Phylogenetic relationships among isolates were visualized using a neighbor-joining tree, which incorporated antimicrobial resistance, virulence, and stress resistance gene profiles as well as gut microbiota taxa relative abundances.

**Data Availability**

All sequencing data have been deposited in the NCBI Sequence Read Archive under BioProject accession numbers PRJNA949542 and PRJNA948843.

**References**

1. Posteraro B, De Maio F, Motro Y, Menchinelli G, De Lorenzis D, Marano RBM, Aljanazreh B, Errico FM, Massaria G, Spanu T, Posteraro P, Moran-Gilad J, Sanguinetti M. In-depth characterization of multidrug-resistant NDM-1 and KPC-3 co-producing *Klebsiella pneumoniae* bloodstream isolates from Italian hospital patients. Microbiol Spectr. 2024;12:e0330523. https://doi.org/10.1128/spectrum.03305-23.
2. Posteraro B, De Maio F, Gasbarrini A. Profiling the gastrointestinal microbiota. Methods Mol Biol. 2021;2283:83–92. https://doi.org/10.1007/978-1-0716-1302-3_10.
3. De Maio F, Ianiro G, Coppola G, Santopaolo F, Abbate V, Bianco DM, Del Zompo F, De Matteis G, Leo M, Nesci A, Nicoletti A, Pompili M, Cammarota G, Posteraro B, Sanguinetti M, Gasbarrini A, Ponziani FR. Improved gut microbiota features after the resolution of SARS‑CoV‑2 infection. Gut Pathog. 2021;13:62. https://doi.org/10.1186/s13099-021-00459-9.
